# Antinuclear Antibodies in Polycystic Ovary Syndrome: A Systematic Review of Observational Studies

**DOI:** 10.3390/ijms26199493

**Published:** 2025-09-28

**Authors:** Jakub Kwiatkowski, Nicole Akpang, Lucja Zaborowska, Artur Ludwin

**Affiliations:** 1 1st Department of Obstetrics and Gynecology, Medical University of Warsaw, 02-015 Warsaw, Poland; s085160@student.wum.edu.pl (J.K.); s084954@student.wum.edu.pl (N.A.); zaborowska.lucja@doctoral.uj.edu.pl (L.Z.); 2 Doctoral School of Medical and Health Sciences, Jagiellonian University Collegium Medicum, 31-530 Cracow, Poland

**Keywords:** polycystic ovary syndrome, PCOS, antinuclear, ANA, autoimmunity, antibodies, antibody

## Abstract

Polycystic ovary syndrome (PCOS), the most common endocrine disorder in reproductive-age women, is characterized by menstrual irregularities and polycystic ovarian morphology. It is also associated with insulin resistance, chronic inflammation, and oxidative stress, all of which may promote autoimmunity. Several studies have suggested a higher occurrence of antinuclear antibodies (ANA) in PCOS but the main challenge in this field is the inconsistency of findings due to heterogeneous study designs and assay methods. However, to date, no systematic review has synthesized this evidence. We conducted a systematic review to evaluate the prevalence and serum levels of ANA in women with PCOS. A comprehensive search was performed in PubMed, Embase, and Scopus, and 13 studies were ultimately included, comprising 924 women with PCOS and 1172 controls. ANA were elevated in about half of the studies, while the remainder found no significant differences between PCOS and controls. Anti-dsDNA antibodies were the most consistently investigated ANA subtype, with most studies reporting higher levels or prevalence in PCOS. For other ANA subtypes, the evidence was limited and inconclusive, largely due to methodological variability across studies. This systematic review suggests that ANA may be elevated in a subset of women with PCOS, but the current evidence remains inconsistent. These findings highlight the need for methodological standardization in ANA assessment to enable clearer conclusions and to clarify whether ANA positivity has clinical relevance in this population.

## 1. Introduction

Polycystic ovary syndrome (PCOS) is an endocrine disorder in women characterized by ovulatory dysfunction and androgen excess [1]. The diagnosis relies on clinical presentation and biochemical assessment and typically follows the Rotterdam criteria: two of three features (ovulatory dysfunction, clinical/biochemical hyperandrogenism, polycystic ovarian morphology/anti-Müllerian hormone elevation) after exclusion of alternative etiologies of observed symptoms such as thyroid dysfunction, hyperprolactinemia, non-classic congenital adrenal hyperplasia, Cushing’s syndrome, or androgen-secreting tumors [2]. In the reproductive-age female population, prevalence generally ranges from 8% to 13% depending on diagnostic criteria, with up to 70% of cases remaining undiagnosed [2,3]. PCOS has a complex, multifactorial etiology involving genetic susceptibility, developmental and environmental influences, insulin resistance, and dysregulation of hypothalamic–pituitary–ovarian signaling [1,4,5,6]. Clinically, it is linked to reproductive difficulties (subfertility/infertility, early pregnancy loss, adverse pregnancy outcomes) [7,8], hyperandrogenic symptoms (hirsutism, acne, alopecia) [9], and cardiometabolic risk (insulin resistance, dyslipidemia, obesity, type 2 diabetes) [10] with substantial psychosocial and quality-of-life impact [11].

These clinical features coexist with chronic low-grade inflammation and immune perturbations in PCOS, which may increase susceptibility to autoimmunity [12,13]. Mechanistically, persistent oxidative stress and dysregulated adaptive immunity could facilitate loss of tolerance and the production of autoantibodies [12,14]. Indeed, several reports have demonstrated elevated levels of various autoantibodies in PCOS, including anti-thyroid peroxidase (anti-TPO) [15], anti-thyroglobulin (anti-TG) [15], antinuclear antibodies (ANA) [16], and anti-ovarian antibodies [17], among others [18]. The detection of such autoantibodies in PCOS may reflect a state of subclinical immune activation [19]. Among them, antinuclear antibodies (ANA) are of particular interest. ANA bind to components of the cell nucleus, including proteins, deoxyribonucleic acid (DNA), ribonucleic acid (RNA), and nucleic acid–protein complexes [20]. In the context of chronic low-grade inflammation, accelerated cellular apoptosis in women with PCOS [21] and increased exposure to intracellular, including nuclear, antigens may increase the likelihood of ANA formation [22,23,24]. If confirmed, elevated ANA in PCOS, considered a hallmark of generalized autoimmune activation [25], would support the concept of autoimmune involvement in PCOS and provide a unifying explanation for the heterogeneous autoantibody findings reported by multiple investigators.

Clinically, ANA serve as serological markers of systemic autoimmune rheumatic diseases (e.g., systemic lupus erythematosus, Sjögren’s syndrome, systemic sclerosis) [26]. Low-titer ANA are also common in otherwise healthy individuals, especially women [27] and older adults [28], and may also occur transiently with intercurrent infections [29], during pregnancy [30], or with certain medications [31]. Accordingly, ANA positivity, particularly at low titers, does not in itself establish disease and must be interpreted in a clinical context [32]. While autoimmune thyroid disease is consistently more prevalent in PCOS [15,33,34], evidence for higher rates of ANA-related systemic autoimmune rheumatic diseases (Sjögren’s syndrome [35], rheumatoid arthritis [36,37], systemic sclerosis [37], undifferentiated connective tissue disease [37]) remains limited. Nevertheless, several single-center studies have reported higher ANA positivity in women with PCOS [16,18], whereas others found no significant difference [38]. This heterogeneity leaves an open question: does PCOS represent another clinical state in which, against a background of low-grade inflammation, ANA occur more frequently? To address this gap, we conducted a systematic review quantifying ANA prevalence and levels in PCOS patients versus non-PCOS controls.

## 2. Methods

A systematic review followed the Preferred Reporting Items for Systematic reviews and Meta-Analyses (PRISMA) 2020 guidelines [39]. The protocol was registered at the International Prospective Register of Systematic Reviews (PROSPERO) (registration number: CRD42024622202) and is available at https://www.crd.york.ac.uk/PROSPERO/view/CRD42024622202 (accessed on 28 August 2025). This review synthesizes evidence on antinuclear autoantibodies in women with PCOS.

### 2.1. Eligibility Criteria

Studies were included if they met the following criteria:

1. Observational design: case–control, cross-sectional, or cohort.
2. Population: reproductive-age women with PCOS.
3. Exposure: prevalence/level of ANA measured by validated methods, such as immunoassays (e.g., enzyme-linked immunosorbent assay (ELISA)) or immunofluorescence (IF).
4. Comparator: women without PCOS from clinical or population samples.
5. Outcomes: (primary) prevalence of ANA positivity and/or levels of ANA and (if reported) associations with clinical/biochemical features of PCOS.

Exclusion criteria included:

1. Pediatric populations (<18 years), pregnancy, or post-menopausal women.
2. Studies primarily enrolling patients with systemic autoimmune rheumatic diseases (e.g., systemic lupus erythematosus, Sjögren’s syndrome, systemic sclerosis, mixed connective tissue disease).
3. Lack of an appropriate comparator group.
4. No ANA-specific results (e.g., only composite “autoantibodies” without ANA breakdown).
5. Book chapters, case reports, commentaries, conference abstracts, editorials, errata, guidelines (statements, consensuses, position papers), letters to editors, notes, protocols, and reviews (systematic reviews, narrative reviews, meta-analyses).
6. Insufficient data reporting to estimate an effect size.
7. Language: publications in languages other than English were excluded.

### 2.2. Search Strategy

Two investigators (J.K. and N.A.) independently searched for relevant studies in databases including PubMed, Scopus, and Embase. Searches were conducted on 9 August 2025 without restrictions on language or publication date. The search strategy (Supplementary Material S1) combined the following search terms in two concept blocks, expanded with synonyms or MeSH terms: “autoantibodies” (together with ANA-specific terms e.g., “antinuclear antibodies”, “ANA”) AND “polycystic ovary syndrome”.

### 2.3. Study Selection

For citation management, all records were exported to EndNote 21 (Clarivate Analytics, Philadelphia, PA, USA) and de-duplicated. Two investigators (J.K. and N.A.) independently screened titles and abstracts against eligibility criteria. Among studies on autoantibodies in PCOS, we identified those that assessed ANA either alone or alongside other autoantibodies. Following primary screening, two authors (J.K. and N.A.) reviewed the full texts of the potentially eligible articles independently based on the inclusion and exclusion criteria. Any conflicts were resolved by a third author (L.Z.).

**Figure 1:**
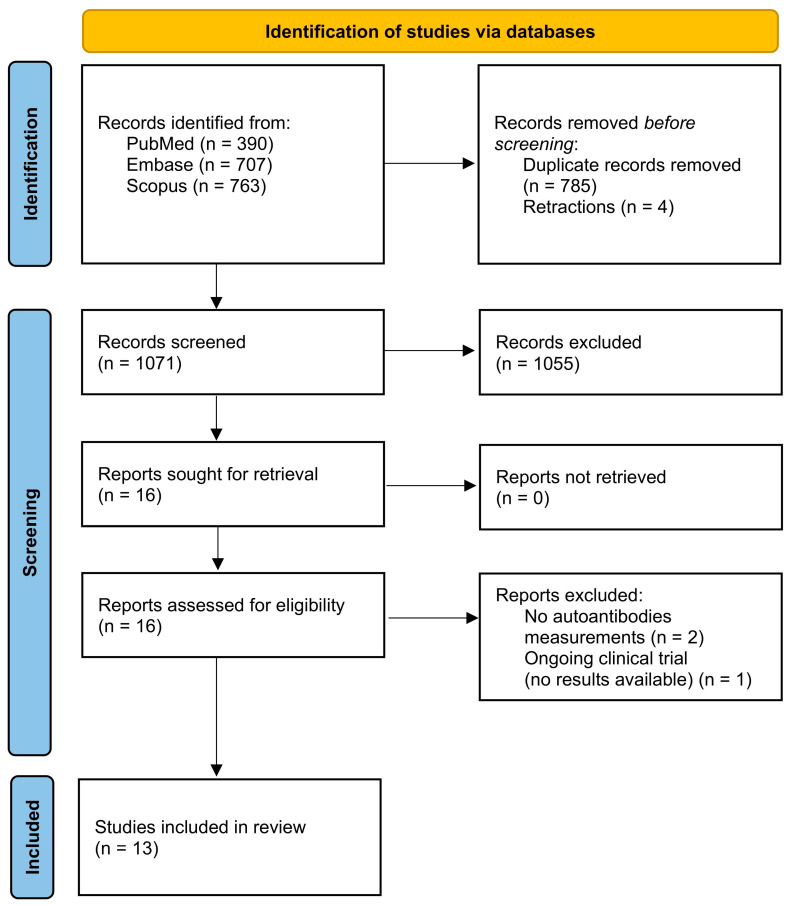
Preferred Reporting Items for Systematic reviews and Meta-Analyses (PRISMA) flow diagram of the study selection process. Adapted from Ref. [39].

The database search yielded 1860 records (PubMed *n* = 390, Embase *n* = 707, Scopus *n* = 763). Before screening, 789 records were removed (785 duplicates; 4 retractions), leaving 1071 records for title and abstract screening. Of these, 1055 were excluded. We sought 16 full-text reports and successfully retrieved all. After full-text assessment, we excluded three reports: two did not include autoantibody measurements [37,40] and one was an ongoing clinical trial without results [41]. Ultimately, 13 studies met the inclusion criteria and were included in the review. The PRISMA flow diagram is presented in Figure 1.

### 2.4. Data Extraction

Two researchers (J.K. and N.A.) independently extracted relevant data from all included studies using a predesigned spreadsheet.

1. Study descriptors: author, year, country, study design.
2. Participants: total number and group sizes, PCOS diagnostic criteria, age and body mass index (BMI), any matching/adjustment for age or BMI, exclusion of autoimmune diseases/systemic autoimmune rheumatic diseases (SARDs), and exposure to medications known to induce ANA or drug-induced lupus.
3. ANA measurement: assay type (e.g., human epithelial type 2 cells indirect immunofluorescence (HEp-2 IIF), ELISA).
4. Outcomes: ANA positivity and ANA serum levels for PCOS and controls, antigen-specific ANA detected on reflex testing, ANA-specific associations with clinical/biochemical features (if reported).

Due to marked methodological heterogeneity across studies, no quantitative meta-analysis was performed. Instead, findings were synthesized qualitatively and presented in structured tables, stratified by antibody type, detection method, and outcome (prevalence vs. serum levels). Missing data were denoted as “ND” (no data). No numerical conversions or imputations were undertaken, with the sole exception that when only a total and a percentage were provided, we derived the corresponding number of cases (rounded to the nearest integer).

### 2.5. Risk of Bias Assessment

Two reviewers (J.K. and N.A.) independently evaluated study quality using a modified Newcastle–Ottawa Scale (NOS) tailored to ANA studies in PCOS; disagreements were resolved by a third reviewer (L.Z.) Detailed scoring criteria are provided in Supplementary Material S2.

- Selection (max 4 points).
  1. PCOS case definition based on recognized criteria (Rotterdam 2003/National Institutes of Health (NIH)/Androgen Excess–Polycystic Ovary Syndrome Society (AE-PCOS)).
  2. Clearly defined and appropriate inclusion and exclusion criteria for cases.
  3. Control group well described and drawn from the same source population as PCOS cases, with appropriate criteria.
  4. Validated ANA assay with a stated positivity threshold.
- Comparability (max 2 points).
  1. Matching or adjustment for age.
  2. Matching or adjustment for BMI.
- Outcome (max 3 points).
  1. Transparent outcome definition and complete reporting in both groups.
  2. Consideration of potential confounders (history of autoimmune diseases/SARDs or exposure to medications known to induce ANA).
  3. Appropriate statistical analysis of the results reported.
- NOS was modified to include explicit criteria for assay definition, positivity thresholds, and for the consideration of key ANA-specific confounders (SARDs or ANA-inducing drugs), making the tool fit for this study.
- Total scores of 7–9 were considered low risk of bias, 4–6 intermediate, and 0–3 high, consistent with thresholds commonly applied in previous NOS-based systematic reviews [42]. In order to explore the potential impact of study quality on the findings, we additionally compared results after excluding studies with intermediate or high risk of bias. This approach allowed us to assess how much the reported heterogeneity could be attributed to lower-quality studies.

## 3. Results

### 3.1. Summary of Included Studies Characteristics

Thirteen studies were ultimately included, and their key features are summarized in Table 1. The evidence base is geographically diverse, comprising one study from Italy; three from Iran; three from Iraq; two from India; and one study each from Austria, Egypt, Estonia, and the Slovak Republic. Study design was provided for most reports. In studies lacking an explicit designation, the design was deduced from the detailed methods (participant selection, timing of measurements, and comparator structure) according to conventional epidemiologic standards. In total, nine studies were case–control, two were prospective controlled clinical studies, and two had a cross-sectional design. For the prospective controlled clinical studies, only baseline ANA levels assessed prior to the interventional procedure were considered in the analysis. For PCOS ascertainment, the 2003 Rotterdam criteria were used in 10 studies [2]. One study applied the NIH criteria [43], one used the AE-PCOS Society criteria [44], and one relied on alternative, pre-Rotterdam definitions [45].

Across the 13 included studies, control cohorts consisted primarily of healthy women without polycystic ovary syndrome. Age matching was reported in 10 studies and in 4 of these, BMI was also matched. Most reports implemented explicit exclusion criteria to strengthen internal validity: seven excluded exposure to antinuclear antibody–inducing or other potentially interfering medications; one excluded chronic illnesses; two excluded drug-induced lupus; and three explicitly excluded autoimmune disease. Thyroid-related restrictions were also common, with two studies enrolling euthyroid controls, two excluding hyperthyroidism, and one excluding any history of thyroid disease. Overall, the comparator groups were well characterized and broadly comparable to the case groups on key confounders.

Across the 13 studies, PCOS group sizes ranged from 20 to 152 and control group sizes from 30 to 392. In total, there were 924 women with PCOS and 1172 controls. Where reported, mean age ranged from 22.67 to 30.23 years in PCOS and 22.84 to 31.00 years in controls. Mean BMI ranged from 23.34 to 28.20 kg/m^2^ in PCOS and 21.31 to 26.10 kg/m^2^ in controls. Several studies did not report age and/or BMI.

Assay methodology varied across the 13 studies. Enzyme-linked immunosorbent assay was used in nine studies, indirect immunofluorescence in two, and one study employed both approaches. In one report, the measurement method was not specified. Overall, the evidence base is predominantly ELISA-based, with a minority of IIF-based assessments.

### 3.2. Risk of Bias in Included Studies

The methodological quality assessment using the modified Newcastle–Ottawa Scale is presented in Table 2. A total of nine studies were categorized as having a low risk of bias (7–9 points), three as having an intermediate risk (4–6 points), and one study as having a high risk of bias (0–3 points).

### 3.3. Main Findings

The prevalence of different ANA autoantibodies was investigated in eight studies (Table 3) [16,18,38,50,51,52,53,55], seven of which evaluated ANA overall screening (three using IIF on tissue [18,38] or HEp-2 [51], three using ELISA [16,50,52], and one not specifying the method [55]). In two of these, ANA-positive samples were further confirmed and subtyped [51,52]. Three studies investigated selected ANA subtypes using ELISA: anti-double-stranded DNA antibodies (anti-dsDNA) [50,51,53], anti-nucleosome antibodies (anti-nucleosome) [53], and anti-histone antibodies (anti-histone) [53].

The serum levels of different ANA autoantibodies were assessed in a total of seven studies (Table 4), all of which used ELISA [46,47,48,49,50,53,54]. Among them, three measured general ANA levels (ANA overall screening) [47,49,50], but only two reported the specific antigen composition of the assay [47,50]. All seven studies also examined selected ANA subtypes: most frequently anti-dsDNA [46,47,48,49,50,53], followed by anti-nucleosome [47,48,53] and anti-histone [47,53,54]. One study assessed several ANA autoantibodies (anti-Sjögren’s-syndrome-related antigen A (anti-SSA), anti-Sjögren’s-syndrome-related antigen B (anti-SSB), anti-Smith antigen (anti-Sm), anti-ribonucleoprotein/Smith (anti-RNP/Sm), anti-DNA topoisomerase I (anti-Scl-70), and anti-histidyl-tRNA synthetase (anti-Jo-1)) within a panel [46].

**Table 3:** Summary of studies on ANA prevalence in PCOS: antibodies, detection methods, and main findings.

Author, Year	Antibody	Detection Method	PCOS Positive *n*/*N* (%)	Control Positive *n*/*N* (%)	*p* Value	Outcome
ANA (overall screening)						
Garelli et al., 2013 [38]	ANA	IIF (rat liver/kidney cryostat sections)	0/113 (0%)	0/100 (0%)	NA	NS
Makled et al., 2015 [50]	ANA	ELISA (SSA, SSB, RNP70, Sm, RNP/Sm, Scl-70, CENP-B, Jo-1)	18/50 (36%)	3/50 (6%)	<0.001	↑ PCOS
Petrikova et al., 2015 [51]	ANA	IIF (HEp-2); ANA+ samples confirmed and subtyped by ELISA (SSA, SSB, RNP)	1/152 (0.7%)	2/74 (2.7%)	0.250	NS
Rashid et al., 2018 [16]	ANA	ELISA (antigens not specified)	16 */89 (18.4%)	2 */87 (2.3%)	<0.01	↑ PCOS
Reimand et al., 2001 [18]	ANA	IIF (rat liver/kidney, mouse stomach, human thyroid cryostat sections)	6/20 (30.0%)	14/392 (3.6%)	<0.005	↑ PCOS
Samsami et al., 2013 [52]	ANA	ELISA (U1-RNP, RNP/Sm, Sm, SSA, SSB, Scl-70, CENP-B, Jo-1); ANA+ samples subtyped	3/35 (8.6%)–all anti-SSA+ (after subtyping)	0/35 (0%)	ND	NS
Shrivastava et al., 2024 [55]	ANA	method not specified (antigens not specified)	7/70 (10.0%)	3/70 (4.3%)	0.326	NS
ANA subtypes						
Makled et al., 2015 [50]	anti-dsDNA	ELISA	14/50 (28%)	1/50 (2%)	<0.001	↑ PCOS
Petrikova et al., 2015 [51]	anti-SSA	ELISA	1/152 (0.7%)	1/74 (1.4%)	0.549	NS
Petrikova et al., 2015 [51]	anti-SSB	ELISA	0/152 (0%)	1/74 (1.4%)	0.327	NS
Petrikova et al., 2015 [51]	anti-RNP	ELISA	0/152 (0%)	0/74 (0%)	NA	NS
Petrikova et al., 2015 [51]	anti-dsDNA	ELISA	3/152 (2%)	0/74 (0%)	0.553	NS
Samsami et al., 2014 [53]	anti-dsDNA	ELISA	8/35 (22.9%)	2/35 (5.7%)	0.004	↑ PCOS
Samsami et al., 2014 [53]	anti-histone	ELISA	4/35 (11.4%)	1/35 (2.9%)	0.051	NS
Samsami et al., 2014 [53]	anti-nucleosome	ELISA	0/35 (0%)	0/35 (0%)	NA	NS

↑ PCOS indicates higher prevalence in women with PCOS. (Antibody)+ indicate (antibody)-positive cases. * Number of cases calculated from total and % (rounded to integer). Antigens in parentheses denote those included in the ANA assay. ANA—antinuclear antibodies; anti-dsDNA—anti-double-stranded DNA antibodies; anti-histone—anti-histone antibodies; anti-nucleosome—anti-nucleosome antibodies; anti-SSA—anti-Sjögren’s-syndrome-related antigen A (SSA/Ro) antibodies; anti-SSB—anti-Sjögren’s-syndrome-related antigen B (SSB/La) antibodies; anti-RNP—anti-ribonucleoprotein (RNP) antibodies; CENP-B—centromere protein B antigen; ELISA—enzyme-linked immunosorbent assay; HEp-2—human epithelial type 2 cells (standard substrate for ANA indirect immunofluorescence assays); IIF—indirect immunofluorescence; Jo-1—histidyl-tRNA synthetase antigen; *n*/*N*—number positive/total tested; NA—not applicable; ND—no data; NS—not significant; PCOS—polycystic ovary syndrome; Scl-70—DNA topoisomerase I antigen; Sm—Smith antigen; U1-RNP—U1 ribonucleoprotein antigen.

**Table 4:** Summary of studies on ANA levels in PCOS: antibodies, detection methods, and main findings.

Author, Year	Antibody	Detection Method	*N* (PCOS/Control)	PCOS Mean ± SD	Control Mean ± SD	*p* Value	Outcome
ANA (overall screening)							
Hefler-Frischmuth et al., 2010 [47]	ANA	ELISA (SSA, SSB, Sm, RNP70, RNP/Sm, Scl-70, CENP-B, Jo-1)	109/109	0.20 ± 0.50 OD	0.18 ± 0.20 OD	0.8	NS
Kakoo et al., 2023 [49]	ANA	ELISA (antigens not specified)	40/40	9.2 * IU/mL	5.9 * IU/mL	0.002	↑ PCOS
Makled et al., 2015 [50]	ANA	ELISA (SSA, SSB, RNP70, Sm, RNP/Sm, Scl-70, CENP-B, Jo-1)	50/50	9.0 ± 6.1 IU/mL	5.4 ± 2.3 IU/mL	<0.001	↑ PCOS
ANA subtypes							
Hamedi et al., 2014 [46]	anti-dsDNA	ELISA	102/100	42.5 ± 38 IU/mL	35.4 ± 39 IU/mL	0.23	NS
Hamedi et al., 2014 [46]	anti-SSA	ELISA	102/100	0.16 ± 0.2 OD	0.16 ± 0.2 OD	0.91	NS
Hamedi et al., 2014 [46]	anti-SSB	ELISA	102/100	0.15 ± 0.6 OD	0.16 ± 0.7 OD	0.37	NS
Hamedi et al., 2014 [46]	anti-Sm	ELISA	102/100	0.15 ± 0.9 OD	0.16 ± 0.8 OD	0.44	NS
Hamedi et al., 2014 [46]	anti-RNP/Sm	ELISA	102/100	0.13 ± 0.06 OD	0.13 ± 0.06 OD	0.59	NS
Hamedi et al., 2014 [46]	anti-Scl-70	ELISA	102/100	0.13 ± 0.05 OD	0.13 ± 0.05 OD	0.93	NS
Hamedi et al., 2014 [46]	anti-Jo-1	ELISA	102/100	0.10 ± 0.05 OD	0.09 ± 0.3 OD	0.47	NS
Hefler-Frischmuth et al., 2010 [47]	anti-histone	ELISA	109/109	7.8 ± 7.7 IU/mL	5.5 ± 6.1 IU/mL	0.02	↑ PCOS
Hefler-Frischmuth et al., 2010 [47]	anti-nucleosome	ELISA	109/109	7.2 ± 8.2 IU/mL	9.4 ± 14.4 IU/mL	0.2	NS
Hefler-Frischmuth et al., 2010 [47]	anti-dsDNA	ELISA	109/109	4.6 ± 3.8 IU/mL	3.8 ± 1.6 IU/mL	0.02	↑ PCOS
Ibrahim et al., 2019 [48]	anti-nucleosome	ELISA	50/50	8.0 ± 2.7 IU/mL	5.1 ± 2.6 IU/mL	<0.001	↑ PCOS
Ibrahim et al., 2019 [48]	anti-dsDNA	ELISA	50/50	54.2 ± 20.3 IU/mL	24.0 ± 15.0 IU/mL	<0.001	↑ PCOS
Kakoo et al., 2023 [49]	anti-dsDNA	ELISA	40/40	ND	ND	0.01	↑ PCOS
Makled et al., 2015 [50]	anti-dsDNA	ELISA	50/50	56.3 ± 25.7 IU/mL	26.0 ± 10.8 IU/mL	<0.001	↑ PCOS
Samsami et al., 2014 [53]	anti-dsDNA	ELISA	35/35	18.1 ± 12.6 IU/mL	ND	0.029	↑ PCOS
Samsami et al., 2014 [53]	anti-histone	ELISA	35/35	8.74 ± 5.7 IU/mL	ND	ND	NS
Samsami et al., 2014 [53]	anti-nucleosome	ELISA	35/35	2.28 ± 1.6 IU/mL	ND	ND	NS
Shaheed et al., 2020 [54]	anti-histone	ELISA	60/30	3.11 ± 0.06 ** IU/mL	1.60 ± 0.11 ** IU/mL	0.0001	↑ PCOS

Numerical values are given as mean ± standard deviation, unless otherwise specified. * Values given as median. ** Values given as standard error of mean (SEM). ↑ PCOS indicates higher prevalence in women with PCOS. Antigens in parentheses denote those included in the ANA assay. ANA—antinuclear antibodies; anti-dsDNA—anti-double-stranded DNA antibodies; anti-SSA—anti-Sjögren’s-syndrome-related antigen A (SSA/Ro) antibodies; anti-SSB—anti-Sjögren’s-syndrome-related antigen B (SSB/La) antibodies; anti-Sm—anti-Smith antigen antibodies; anti-RNP/Sm—anti-ribonucleoprotein/Smith antibodies; anti-Scl-70—anti-DNA topoisomerase I antibodies; anti-Jo-1—anti-histidyl-tRNA synthetase antibodies; anti-nucleosome—anti-nucleosome antibodies; anti-histone—anti-histone antibodies; CENP-B—centromere protein B; ELISA—enzyme-linked immunosorbent assay; IU/mL—international units per milliliter; OD—optical density; ND—no data; NS—not significant; *N*—number of participants; PCOS—polycystic ovary syndrome; RNP70—ribonucleoprotein 70; SD—standard deviation.

#### 3.3.1. ANA Overall Screening

Studies on ANA overall screening, although relatively numerous, were highly heterogeneous in both methodology and results.

- Of the seven prevalence studies [16,18,38,50,51,52,55], three found higher prevalence in women with PCOS [16,18,50], while four found no differences between groups [38,51,52,55].
- Regarding ANA levels, two studied reported higher levels in PCOS [49,50], whereas another found no differences [47].

Among the seven prevalence studies, three used the IIF method but with different substrates (various animal tissues, HEp-2 cells) [18,38,51]. In one study no ANA-positive samples were observed in either group [38], while on the contrary in another ANA prevalence was significantly higher in PCOS women (6/20, 30%) [18]. The HEp-2–based study reported very low prevalence overall (1/152, 0.7% PCOS vs. 2/74, 2.7% controls), without significant differences [51]. Notably, both of the studies with these most extreme findings were judged to have moderate [38] or high risk of bias [18] according to the NOS assessment, which limits the reliability of their conclusions and complicates data interpretation.

Three studies used ELISA and yielded inconsistent results [16,50,52]. One was limited by a small sample size (3/35 PCOS ANA+, 0/35 controls) and was likewise rated as having moderate risk of bias [52], while the other with a larger cohort found a statistically significant difference (16/89, 18.4% PCOS ANA+ vs. 2/87, 2.3% controls) [16]. Makled et al. likewise demonstrated a significantly higher prevalence of ANA in PCOS (18/50, 36% vs. 3/50, 6%; *p* < 0.001) [50]. One additional study did not report the assay method and found no significant difference (7/70 vs. 3/70) [55].

Taken together, subgrouping across studies shows that methodological differences are the main source of heterogeneity in ANA prevalence findings. IIF results diverged depending on the substrate: rat kidney/liver yielded 0% positivity [38], mixed human/animal tissues gave 30% [18], and HEp-2 cells produced uniformly low rates (0.7% vs. 2.7%) [51]. ELISA studies showed a more consistent signal in the larger cohorts, with prevalence between 18% and 36% in PCOS compared with 2–6% in controls [16,50], whereas the smaller moderate-risk study reported only 8.6% (3/35) positives in PCOS and none in controls [52].

When results are viewed by study quality, both low- and high-risk studies generated extreme findings: low-risk cohorts ranged from very low ANA positivity (0.7%) [51] to the highest reported prevalence of 36% [50], with others showing intermediate values around 10–18% [16,55], while the high-risk studies reported both 0% [38] and 30% [18]. The single moderate-risk study showed a low prevalence of 8.6% [52]. This pattern indicates that heterogeneity was present across all quality categories and is more likely driven by assay methodology, sample size, or population differences.

For ANA levels, three studies using ELISA reported conflicting results [47,49,50]. One study with moderate risk of bias reported higher ANA levels in PCOS but presented only medians (9.2 vs. 5.9 international units per milliliter (IU/mL)) without interquartile range (IQR) (shown only graphically) and did not specify the antigen composition of the assay [49]. Another study, despite assessing a wide range of antigens (Sjögren’s-syndrome-related antigen A (SSA), Sjögren’s-syndrome-related antigen B (SSB), Smith antigen (Sm), ribonucleoprotein 70 (RNP70), ribonucleoprotein/Smith complex antigen (RNP/Sm), DNA topoisomerase I (Scl-70), centromere protein B (CENP-B), histidyl-tRNA synthetase (Jo-1)), found no significant differences (0.20 ± 0.50 vs. 0.18 ± 0.20 optical density (OD)), although it reported more frequent occurrence of some specific subtypes (anti-histone and anti-dsDNA, see below) [47]. By contrast, Makled et al. demonstrated significantly higher ANA levels in PCOS compared to controls (9.0 ± 6.1 vs. 5.4 ± 2.3 IU/mL, *p* < 0.001) [50]. These results are also difficult to compare directly due to the use of different measurement units (IU/mL vs. OD).

Overall, heterogeneity in ANA level findings appears to arise primarily from methodological differences. While both moderate- [49] and low-risk [50] studies reported higher levels, results were inconsistent and reported in non-comparable formats (IU/mL vs. OD, medians vs. means). The most robust low-risk study [50] found significantly higher ANA levels in PCOS, whereas another [47] showed no differences, underscoring the need for standardized assays and reporting.

It should also be noted that in one study [48], the authors referred to “ANA” in the text, although the methodology clearly indicated measurement of anti-nucleosome antibodies levels. This further illustrates the heterogeneity in terminology and assay reporting across studies.

Taken together, the available evidence on ANA screening in PCOS is inconsistent, with about half of the studies reporting increased prevalence or levels and the remainder finding no significant differences, with possible reporting biases.

#### 3.3.2. Anti-dsDNA

Anti-dsDNA antibodies were the most frequently investigated ANA subtype across the included studies. Six studies assessed serum anti-dsDNA levels [46,47,48,49,50,53], five of which reported significantly higher concentrations in women with PCOS compared with controls [47,48,49,50,53], while one study found no significant differences [46]. Notably, five of these studies were judged to be at low risk of bias [46,47,48,50,53]. Despite this consistent direction, the magnitude of differences varied considerably: for example, Ibrahim et al. reported markedly elevated levels (54.2 ± 20.3 vs. 24.0 ± 15.0 IU/mL) [48], whereas Hefler-Frischmuth et al. observed only modest differences (4.6 ± 3.8 vs. 3.8 ± 1.6 IU/mL) [47]. In Kakoo et al., results were presented only graphically, without exact numerical values, and thus only the authors’ conclusion of significantly higher anti-dsDNA levels in PCOS could be extracted [49]. Similarly, in Samsami et al. (2014), baseline anti-dsDNA was reported as significantly higher in PCOS compared with controls (*p* = 0.029), but absolute control values were not provided [53]. All studies applied ELISA-based assays and reported anti-dsDNA in standardized IU/mL, which limits the scope for methodological subgrouping and makes these results more internally consistent than ANA screening. Likewise, almost all studies were judged to be at low risk of bias, so study quality does not explain the variability. Instead, residual heterogeneity likely arises from differences in ELISA kits and positivity thresholds, as well as variation in sample size and reporting practices.

Prevalence of anti-dsDNA positivity was reported in three studies [50,51,53], two of which demonstrated significantly higher prevalence in PCOS [50,53]. Again, the reported prevalence varied widely despite consistent use of ELISA, ranging from 28% (14/50 PCOS vs. 1/50 controls) [50] to only 2% (3/152 PCOS vs. 0/74 controls) [51].

Overall, most studies point toward increased anti-dsDNA levels or prevalence in PCOS, but heterogeneity in assays, reporting strategies, and incomplete data presentation limit the comparability.

#### 3.3.3. Anti-Nucleosome

Anti-nucleosome antibodies were evaluated in three studies, all using ELISA with low risk of bias [47,48,53]. Results were heterogeneous. One study [48], which initially referred to “ANA” but in fact measured anti-nucleosome antibodies, reported significantly higher levels in women with PCOS compared to controls (8.0 ± 2.7 vs. 5.1 ± 2.6 IU/mL, *p* < 0.001). In contrast, two other studies found no significant differences between groups, with mean levels being similar in PCOS and control women [47,53]. In the study by Samsami et al. (2014), although results for PCOS patients were provided (2.28 ± 1.6 IU/mL), the authors did not report baseline values for the control group, which limits the interpretability of these findings [53].

With regard to prevalence, only Samsami et al. (2014) addressed this outcome, reporting that all PCOS patients and controls were negative for anti-nucleosome antibodies [53].

Overall, the available evidence on anti-nucleosome antibodies in PCOS is inconclusive: while one study demonstrated elevated levels, most others found no differences, and prevalence data do not support an association.

#### 3.3.4. Anti-Histone

Anti-histone antibodies were investigated in three studies assessing serum levels and in one study reporting prevalence, all with low risk of bias [47,53,54]. Two of the level studies found significantly higher concentrations of anti-histone antibodies in women with PCOS compared to controls (3.11 ± 0.06 vs. 1.60 ± 0.11 IU/mL, *p* = 0.0001; 7.8 ± 7.7 vs. 5.5 ± 6.1 IU/mL, *p* = 0.02) [47,54]. In contrast, one smaller study reported similar anti-histone levels between groups (8.74 ± 5.7 IU/mL in PCOS vs. not reported for controls), with no significant difference [53]. Regarding prevalence, Samsami et al. (2014) observed ANA subtyping results where 11.4% of women with PCOS and 2.9% of controls tested positive for anti-histone, but this difference did not reach statistical significance (*p* = 0.051) [53].

Taken together, the evidence suggests that anti-histone antibodies may be elevated in PCOS, although results are inconsistent, with significant associations seen in two larger cohorts but not confirmed in the smaller study.

#### 3.3.5. Other ANA

Three studies assessed additional ANA subtypes, including anti-SSA, anti-SSB, anti-Sm, anti-RNP/Sm, anti-Scl-70, anti-Jo-1 and anti-CENP-B by ELISA panels [46,51,52]. Their occurrence was generally very rare and not consistently different between PCOS and controls. In the study by Samsami et al. (2013) [52], three baseline ANA-positive PCOS cases (8.6%, vs. 0% in controls) were identified, and all were confirmed as anti-SSA-positive upon subtyping, whereas all other subtypes (anti-SSB, anti-Sm, RNP, anti-Scl-70, anti-Jo-1, anti-CENP-B) were negative. Hamedi et al. analyzed ANA subtypes in a quantitative ELISA panel but found no significant differences in antibody levels between groups [46]. Petrikova et al. assessed ANA prevalence using IIF with ELISA confirmation and reported only sporadic positives (e.g., 1/152 anti-SSA in PCOS, 1/74 anti-SSA and 1/74 anti-SSB in controls), without significant group differences [51].

Taken together, apart from dsDNA, nucleosome, and histone antibodies, other ANA subtypes are infrequently detected in PCOS, and no consistent association with PCOS has been demonstrated.

#### 3.3.6. ANA-Associated Factors

Across studies reporting factors associated with ANA levels or specific ANA sub-classes in PCOS, findings clustered in hormonal, inflammatory, and clinical domains (Table 5). Higher serum thyroid-stimulating hormone (TSH) was reported alongside increased ANA measures [47] and was also associated with both anti-nucleosome and anti-dsDNA levels [48]. In the same study, anti-nucleosome levels correlated with higher follicle-stimulating hormone (FSH) and luteinizing hormone (LH), while anti-dsDNA levels correlated with higher FSH [48]. Anti-histone levels were reported together with higher LH, higher concentrations of the proinflammatory cytokines interleukin 17 (IL-17) and interleukin 23 (IL-23), increased sirtuin-1, and the presence of anti-ovarian antibodies [54]. Clinically, anti-Sm positivity was associated with hirsutism [46]. The remaining studies did not report associated factors with ANA levels.

Across studies assessing the prevalence of ANA in PCOS, correlates were reported only in a few datasets. One study linked ANA positivity with clinical and biochemical hyperandrogenism and adverse glycemic measures—reporting co-occurrence with acne vulgaris, higher Ferriman–Gallwey hirsutism scores, higher total testosterone, higher fasting glucose, and higher post-oral glucose tolerance test (OGTT) glucose [16]. Another study reported that ANA positivity coincided with anti-thyroid peroxidase antibody (anti-TPO) positivity [55]. The remaining studies did not report associated factors with ANA prevalence or explicitly stated that none were available.

**Table 5:** Reported associations between ANA (levels or prevalence) and clinical/biochemical factors in women with PCOS.

Author, Year	Antibody (Level/Prevalence)	Associated Factor(s)
Hamedi et al., 2014 [46]	anti-Sm (level)	hirsutism (+)
Hefler-Frischmuth et al., 2010 [47]	ANA (level)	TSH (+)
Ibrahim et al., 2019 [48]	anti-nucleosome (level)	FSH (+), LH (+), TSH (+)
Ibrahim et al., 2019 [48]	anti-dsDNA (level)	FSH (+), TSH (+)
Rashid et al., 2018 [16]	ANA (prevalence)	acne vulgaris (+), FG score (+), total testosterone (+), fasting glucose (+), post-OGTT glucose (+)
Shaheed et al., 2020 [54]	anti-histone (level)	LH (+), IL-17 (+), IL-23 (+), sirtuin-1 (+), AOA (+)
Shrivastava et al., 2024 [55]	ANA (prevalence)	anti-TPO (+)

(+) indicates a positive correlation or association. ANA—antinuclear antibodies; anti-Sm—anti-Smith antigen antibodies; anti-dsDNA—anti-double-stranded DNA antibodies; anti-nucleosome—anti-nucleosome antibodies; anti-histone—anti-histone antibodies; anti-TPO—anti-thyroid peroxidase antibodies; FG score—Ferriman–Gallwey score; LH—luteinizing hormone; FSH—follicle-stimulating hormone; TSH—thyroid-stimulating hormone; OGTT—oral glucose tolerance test; IL-17—interleukin 17; IL-23—interleukin 23; AOA—anti-ovarian antibodies.

A summary of study findings on the prevalence and serum levels of ANA and their subtypes in women with PCOS, based on all eligible observational studies, is presented in Table 6.

## 4. Discussion

This systematic review is the first study to comprehensively evaluate the occurrence of ANA in the population of women with PCOS. Although original studies suggesting a higher prevalence of these autoantibodies in PCOS compared with the general population have been published over the years, no prior work has systematically synthesized the available evidence.

When focusing on general ANA screening, the results across studies were highly heterogeneous. The prevalence of ANA in women with PCOS ranged from as low as 0% [38] to as high as 36% [50], whereas in control groups it ranged from 0% [38,52] to 6% [50]. However, much of this variability appears to be driven by methodological differences and, to a lesser extent, by study quality. When studies with high or moderate risk of bias were excluded [18,38,52], the range slightly narrowed to 0.7–36.0% in PCOS and 2.3–6.0% in controls. Furthermore, when restricting the analysis to studies using ELISA (thereby excluding IIF [18,38,51], studies with unspecified methodology [55], and those with high/moderate risk of bias [18,38,52]), the prevalence range became more consistent, at 18.4–36.0% in PCOS and 2.3–6.0% in controls. This illustrates that heterogeneity in ANA prevalence is largely attributable to methodological and quality-related factors. Overall, roughly half of the studies reported increased ANA prevalence [16,18,50] or serum levels [49,50] in PCOS, while the remainder did not demonstrate significant differences [38,47,51,52,55], most likely resulting from methodological variability. Taken together, these findings suggest that ANA may indeed be elevated in at least a subset of women with PCOS.

In contrast, results regarding specific ANA subtypes—particularly anti-dsDNA antibodies—were more consistent. Anti-dsDNA was the most frequently assessed subtype. Although absolute levels varied considerably across studies (with mean values ranging from 4.6 [47] to 56.3 IU/mL [50] in PCOS and from 3.8 [47] to 35.4 IU/mL [46] in controls), the overall trend strongly suggests an association between PCOS and elevated anti-dsDNA antibodies.

For other subtypes, the evidence is more limited and inconsistent. Anti-nucleosome [47,48,53] and anti-histone antibodies [47,53,54] were each assessed in three studies, with some reporting higher levels in PCOS but others finding no differences. Additional ANA subtypes (SSA, SSB, Sm, RNP, Scl-70, Jo-1, CENP-B) were only rarely investigated, with positive cases occurring sporadically and without reproducible group differences [46,51,52].

From a pathophysiological perspective, the presence of higher ANA levels in PCOS could be explained through several complementary mechanisms: increased availability of nuclear autoantigens due to enhanced apoptosis, oxidative damage, and extracellular DNA release; genetic and epigenetic predisposition favoring aberrant autoantigen presentation; and immune cell imbalances with exaggerated B cell activity, resulting in increased immunoglobulin production and autoantibody generation.

The syndrome is characterized by low-grade chronic inflammation, oxidative stress, hormonal imbalance, and relative hyperestrogenism [56,57,58], all of which may contribute to tissue damage and the subsequent release of intracellular antigens, ultimately triggering ANA production [12]. Recent evidence further highlights the role of oxidative stress in immune activation. Vale-Fernandes et al. (2024) demonstrated that elevated anti-Müllerian hormone (AMH) levels correlate with increased oxidative stress within the follicular microenvironment in PCOS, and that circulating AMH may serve as a surrogate biomarker of this process [59]. This oxidative–immune interplay is of particular interest because it links a routine reproductive biomarker with pathways of autoantibody production, and suggests that combined markers such as AMH and ANA could potentially help identify women at higher risk of adverse reproductive outcomes or autoimmune activation. Importantly, several studies have demonstrated increased apoptosis of ovarian granulosa cells in PCOS [60], accompanied by upregulation of pro-apoptotic markers (Bax, Caspase-3) and downregulation of anti-apoptotic Bcl-2, as well as activation of inflammatory NF-κB pathways, shown in animal and cellular models of PCOS [21,60]. This excessive apoptosis within ovarian follicles provides a local source of nuclear antigens, which may become immunogenic if not efficiently cleared [21,23]. Furthermore, neutrophil extracellular traps (NETs) and elevated circulating DNA have been detected in PCOS serum and follicular fluid, offering a direct route for nuclear autoantigen exposure and subsequent autoantibody production [24].

In addition, PCOS has been associated with a higher frequency of certain class II Human Leukocyte Antigen (HLA) allelic variants (e.g., *HLA-DR* and *HLA-DQ*) [61,62], or expression changes in class I *HLA-B* and *HLA-F* [63], which are also linked to autoimmune diseases, suggesting a possible genetic predisposition to autoimmunity in this population [40]. Since HLA molecules are central to antigen presentation [64], such alterations may facilitate the display of nuclear self-antigens released in PCOS, thereby lowering the threshold for autoantibody production, including ANA. Interestingly, *HLA-DRB1* and *HLA-DQB1* alleles have been reported both in association with PCOS [61] and with ANA positivity in independent cohorts [65]. Although the specific alleles differ across studies and populations, this convergence on the same HLA loci suggests that genetic variation within *DR*/*DQ* may influence aberrant presentation of nuclear antigens, thereby providing a mechanistic link between PCOS susceptibility and ANA production.

Beyond these factors, the detection of increased ANA and related autoantibodies in PCOS may reflect an exaggerated or dysregulated immune response [66], supporting the hypothesis that autoimmunity could play a contributory role in the syndrome’s pathogenesis [4,67,68]. Free light chains of immunoglobulins have been found to be elevated in the blood plasma of women with PCOS, providing direct evidence of enhanced overall antibody production and B cell activation in this condition [69]. Immunophenotyping studies further demonstrate characteristic alterations in immune cell populations in PCOS. These include increased frequencies of activated B cells and higher circulating levels of B cell-activating factor (BAFF), both of which favor antibody and autoantibody production [70,71]. In parallel, a shift in T cell subsets has been observed, with reduced regulatory T cells (Treg) and increased proinflammatory type 1 T helper (Th1) and type 17 T helper (Th17) cells, creating an environment permissive for autoreactive B cell help [72]. Natural killer (NK) cells are often elevated and display enhanced cytotoxicity, potentially contributing to increased cell death and autoantigen release [73]. Moreover, monocytes and macrophages in PCOS preferentially polarize toward a proinflammatory M1 phenotype, secreting interleukin 6 (IL-6) and tumor necrosis factor α (TNF-α), cytokines that further amplify B cell activation and Th17 differentiation [74]. Taken together, these cellular abnormalities provide a coherent explanation for the enhanced humoral immune activation in PCOS and the increased likelihood of ANA production.

From a clinical perspective, it is important to underline that higher ANA titers or a higher prevalence of ANA positivity in PCOS does not necessarily imply a high frequency of systemic autoimmune rheumatologic diseases in this population [27]. Although three of the included studies reported ANA prevalence rates as high as 20–30% in women with PCOS [16,18,50], studies assessing autoimmune comorbidities suggest only a modest numerical increase in certain conditions, such as rheumatoid arthritis or systemic sclerosis, with low prevalence rates of 1–2% in PCOS compared with 1% or less in controls [35,36,37]. Autoimmune connective tissue diseases remain relatively rare in PCOS, and the observed increase in ANA positivity likely reflects immunological activation rather than manifest autoimmune disease. Moreover, ANA positivity can also be detected in several non-pathological conditions, including physiological states such as pregnancy [30] or aging [28], and in otherwise healthy individuals without clinical autoimmune disease [27,32], as well as in other endocrine and gynecological disorders such as endometriosis [75] and autoimmune thyroid disease [76]. Awareness of this association may therefore help clinicians avoid overdiagnosis or unnecessary concern when incidental ANA positivity is identified in PCOS patients [32]. Thus, ANA positivity in PCOS should be interpreted primarily as a marker of non-specific immune activation, rather than evidence of underlying systemic autoimmune disease. This highlights the need for prospective longitudinal studies to determine whether ANA positivity predicts future autoimmune morbidity or simply reflects the background of chronic low-grade inflammation characteristic of PCOS. At present, to our knowledge, no long-term cohort has assessed the persistence of ANA over time or its progression to clinically manifest autoimmune disease in PCOS. Such studies are essential to distinguish transient, inflammation-related positivity from a stable autoimmune trait. Moreover, integrating emerging biomarkers, such as AMH as a surrogate of oxidative stress [59], into longitudinal designs could clarify whether oxidative–immune interactions contribute to sustained autoantibody production and adverse reproductive or metabolic outcomes.

To sum up, the current evidence does not support routine ANA screening in all women with PCOS, as ANA positivity most often reflects non-specific immune activation rather than systemic autoimmune disease. However, testing may be clinically relevant in selected subgroups, such as patients presenting with rheumatologic symptoms or women with recurrent pregnancy loss or implantation failure during assisted reproduction, where ANA positivity has been associated with adverse outcomes in some studies [77,78,79]. From a management perspective, the detection of ANA in PCOS should not prompt routine therapeutic interventions in asymptomatic patients, but it may inform closer monitoring, timely referral for rheumatologic evaluation, or individualized reproductive counseling in women at higher clinical risk.

This systematic review has several important limitations that should be considered when interpreting the findings. The most important relates to the high variability in methodologies used to detect ANA: some studies applied indirect immunofluorescence (IIF) on different substrates (e.g., HEp-2 cells vs. animal tissues), while others employed ELISA assays with differing antigen panels. An additional concern is that assay performance characteristics varied markedly across studies. IIF is considered the reference method for ANA detection, but its sensitivity and specificity depend heavily on the substrate used and the positivity threshold. Lower cut-offs increase sensitivity but reduce specificity, yielding more incidental low-titer positives, whereas higher cut-offs may miss clinically relevant cases. Similarly, ELISA assays differ in the antigen panels employed, with some targeting broad nuclear extracts and others focusing on selected antigens, which can yield divergent prevalence estimates. Additionally, positivity thresholds for ELISA were inconsistent, making direct comparisons unreliable. Moreover, the results lacked consistency in reporting: some studies presented mean values, others reported medians without interquartile ranges, and in some cases, results were shown only graphically. Direct comparisons were also hampered by the use of different measurement units (IU/mL vs. optical density). Inconsistencies in terminology were also evident, with certain studies referring to “ANA” when in fact only specific subtypes (e.g., anti-nucleosome antibodies) were measured, further complicating synthesis of the evidence. In addition, there were limitations inherent to PCOS research in general: diagnostic criteria for PCOS were not uniform across studies, ranging from NIH to Rotterdam definitions and local adaptations, thereby contributing to clinical heterogeneity. The studies also varied in inclusion and exclusion criteria, and only a few applied BMI matching between groups. Overall, the risk of bias across studies was mostly low, but several studies with moderate or high risk were identified, and these tended to present the most heterogeneous and extreme results (e.g., ANA prevalence of 0% in one study and 30% in another), further complicating data synthesis.

Nevertheless, this systematic review highlights several important insights. To our knowledge, it is the first systematic review to comprehensively evaluate the occurrence of ANA and their subtypes in women with PCOS, thereby filling an important gap in the literature. In contrast to earlier narrative reports, we considered both general ANA screening and specific ANA subtypes (including anti-dsDNA, anti-nucleosome, and anti-histone antibodies), which provides a more complete overview of potential autoimmune involvement in PCOS. By separately analyzing studies reporting prevalence and those reporting quantitative serum levels, the review offers a nuanced understanding of how autoantibody patterns differ across methodologies. A further strength is the critical appraisal of methodological aspects, including differences in diagnostic criteria for PCOS, assay platforms (IIF vs. ELISA), and units of measurement, which helps to explain the observed heterogeneity and highlights the need for standardization in future research.

## 5. Conclusions

This systematic review indicates that ANA positivity and elevated ANA subtypes, particularly anti-dsDNA, may occur more frequently in women with PCOS than in controls. While these findings suggest a link between PCOS and increased ANA production, their clinical implications remain uncertain. At present, ANA positivity should not prompt routine screening or management changes in PCOS, and further standardized research, including prospective longitudinal studies, is required before these observations can be meaningfully translated into clinical practice. The evidence remains inconsistent due to methodological heterogeneity and variable study quality. ANA positivity in PCOS should be viewed as a marker of non-specific immune activation rather than systemic autoimmune disease. Standardized assays and prospective studies with long-term follow-up are needed to clarify clinical relevance.

## Figures and Tables

**Table 1:** Characteristics of the studies included in the systematic review investigating the prevalence and levels of antinuclear autoantibodies in women with polycystic ovary syndrome.

Author, Year	Country	Type of Study	Control Group	PCOS Criteria	PCOS			Control			Antibody Measurement Method
					*N*	Age	BMI	*N*	Age	BMI	
Garelli et al., 2013 [38]	Italy	case–control	Healthy women (no history of thyroid disease)	2003 Rotterdam criteria	113	24 ± 6.3	ND	100	27.1 ± 1.2	ND	IIF
Hamedi et al., 2014 [46]	Iran	case–control	Age-, BMI-matched healthy women (no history of ANA-inducing drugs)	AE-PCOS	102	23.6 ± 4.3	23.34 ± 3.8	100	24.24 ± 4.2	22.82 ± 3.6	ELISA
Hefler-Frischmuth et al., 2010 [47]	Austria	case–control	Age-matched healthy, fertile women	NIH	109	29.5 ± 5.1	28.1 ± 6.8	109	30 ± 5.2	ND	ELISA
Ibrahim et al., 2019 [48]	Iraq	case–control	Age-, BMI-matched fertile women without PCOS (no history of ANA-inducing drugs or aromatase inhibitors, hyperthyroidism, or drug-induced lupus)	2003 Rotterdam criteria	50	25.3 ± 2.4	26.3 ± 2.3	50	26.2 ± 3.5	26.1 ± 1.9	ELISA
Kakoo et al., 2023 [49]	Iraq	case–control	Age-matched healthy, fertile women (no chronic illnesses, no drugs)	2003 Rotterdam criteria	40	25.5	26.2	40	ND	ND	ELISA
Makled et al., 2015 [50]	Egypt	case–control	Age-matched fertile women without PCOS (no history of ANA-inducing drugs or aromatase inhibitors, hyperthyroidism, or drug-induced lupus)	2003 Rotterdam criteria	50	25.4 ± 4.69	28.2 ± 1.3	50	27.08 ± 4.57	24.2 ± 0.9	ELISA
Petrikova et al., 2015 [51]	Slovak Republic	case–control	Age-matched healthy women (no history of autoimmune disease)	2003 Rotterdam criteria	152	30.23 ± 6.7	28.08 ± 6.91	74	29.0 ± 4.0	21.31 ± 3.05	IIF; ELISA
Rashid et al., 2018 [16]	India	cross-sectional	Age-matched women without PCOS (no history of systemic disorders or ANA-inducing drugs, or aromatase inhibitors)	2003 Rotterdam criteria	88	22.67 ± 5.53	24.21 ± 4.56	87	22.84 ± 3.64	21.79 ± 3.9	ELISA
Reimand et al., 2001 [18]	Estonia	case–control	Women without PCOS	Other (pre-Rotterdam)	20	ND	ND	392	31	ND	IIF
Samsami et al., 2013 [52]	Iran	Prospective controlled clinical study *	Euthyroid, fertile healthy women	2003 Rotterdam criteria	35	ND	ND	35	ND	ND	ELISA
Samsami et al., 2014 [53]	Iran	Prospective controlled clinical study *	Age-matched euthyroid, fertile healthy women	2003 Rotterdam criteria	35	30.1 ± 2.3	ND	35	29.9 ± 3.1	ND	ELISA
Shaheed et al., 2020 [54]	Iraq	case–control	Age-, BMI-matched healthy women (no history of autoimmune diseases or interfering drugs)	2003 Rotterdam criteria	60	25.25 ± 0.63	24.26 ± 0.39	30	25.60 ± 0.96	24.80 ± 0.51	ELISA
Shrivastava et al., 2024 [55]	India	cross-sectional	Age-, BMI-matched women without PCOS (no history of autoimmune diseases or interfering drugs, including ANA-inducing drugs)	2003 Rotterdam criteria	70	24.90 ± 4.24	23.74 ± 4.78	70	25.75 ± 4.75	22.73 ± 3.88	method not specified

* In prospective controlled clinical studies, only baseline ANA levels measured prior to the intervention were considered for this review. Numerical values are given as mean ± standard deviation. AE-PCOS—Androgen Excess–Polycystic Ovary Syndrome Society criteria; ANA—antinuclear antibodies; BMI—body mass index; ELISA—enzyme-linked immunosorbent assay; IIF—indirect immunofluorescence; *N*—number of participants; ND—no data; NIH—National Institutes of Health criteria; PCOS—polycystic ovary syndrome.

**Table 2:** Quality assessment of the included studies using the modified Newcastle–Ottawa Scale.

Author, Year	Selection				Comparability	Outcome			Sum
	PCOS Definition	Inclusion/Exclusion Criteria	Control Group	Antibody Measurement	Confounders	Clear Results	Antibodies Influencing Factors	Statistical Analysis	
Garelli et al., 2013 [38]	★	★	★	★					4
Hamedi et al., 2014 [46]	★	★		★	★★	★	★	★	8
Hefler-Frischmuth et al., 2010 [47]	★	★	★	★	★	★		★	7
Ibrahim et al., 2019 [48]	★		★	★	★★	★	★	★	8
Kakoo et al., 2023 [49]	★				★		★	★	4
Makled et al., 2015 [50]	★	★	★	★	★	★	★	★	8
Petrikova et al., 2015 [51]	★	★	★	★	★	★	★	★	8
Rashid et al., 2018 [16]	★	★	★		★	★	★	★	7
Reimand et al., 2001 [18]				★		★		★	3
Samsami et al., 2013 [52]	★		★	★		★		★	5
Samsami et al., 2014 [53]	★	★	★	★	★	★		★	7
Shaheed et al., 2020 [54]	★		★	★	★★	★	★	★	8
Shrivastava et al., 2024 [55]	★	★	★		★★	★	★	★	8

Stars indicate points awarded according to the modified Newcastle–Ottawa Scale. The maximum number of stars is four for Selection, two for Comparability, and three for Outcome. The “Sum” column represents the total number of stars assigned (maximum score: 9). PCOS—polycystic ovary syndrome.

**Table 6:** Summary of study findings on the prevalence and serum levels of ANA and their subtypes in women with polycystic ovary syndrome (PCOS).

Autoantibody	Outcome	Direction of Findings in PCOS vs. Controls (Studies Reporting/Total)	Summary of Study Results
ANA (overall screening)			
ANA	Prevalence	↑ (3/7), ↔ (4/7)	Findings are mixed, with roughly half of the studies indicating higher prevalence or levels in PCOS, reflecting substantial methodological heterogeneity.
	Levels	↑ (2/3), ↔ (1/3)	
ANA subtypes			
Anti-dsDNA	Prevalence	↑ (2/3), ↔ (1/3)	This ANA subtype shows the most consistent elevation in PCOS.
	Levels	↑ (5/6), ↔ (1/6)	
Anti-nucleosome	Prevalence	↔ (1)	Reported only in isolated studies, with inconsistent results and insufficient evidence for conclusions.
	Levels	↑ (1/3), ↔ (2/3)	
Anti-histone	Prevalence	↔ (1)	Examined only in some studies, suggesting possible increases but insufficient for firm conclusions.
	Levels	↑ (2/3), ↔ (1/3)	
Other subtypes	Prevalence/Levels	↔/inconclusive	Very rarely assessed, and when tested, detection rates in PCOS were low and without differences from controls.

↑ indicates higher prevalence or levels, whereas ↔ indicates no difference between PCOS and controls. Numbers in parentheses denote the number of studies reporting that finding out of the total number of studies assessing that outcome (e.g., ↑ (1/3) = higher in 1 of 3 studies). ANA—antinuclear antibodies; anti-dsDNA—anti-double-stranded DNA antibodies; anti-histone—anti-histone antibodies; anti-nucleosome—anti-nucleosome antibodies; PCOS—polycystic ovary syndrome.

## Data Availability

The raw data supporting the conclusions of this article will be made available by the authors on request.

## Supplementary Materials

The following supporting information can be downloaded at: https://www.mdpi.com/article/10.3390/ijms26199493/s1.

## Abbreviations

The following abbreviations are used in this manuscript:

AE-PCOS	Androgen Excess–Polycystic Ovary Syndrome Society
AMH	Anti-Müllerian hormone
ANA	Antinuclear antibodies
anti-CENP-B	Anti-centromere protein B antibodies
anti-dsDNA	Anti-double-stranded DNA antibodies
anti-histone	Anti-histone antibodies
anti-Jo-1	Anti-histidyl-tRNA synthetase antibodies
anti-nucleosome	Anti-nucleosome antibodies
anti-RNP/Sm	Anti-ribonucleoprotein/Smith antibodies
anti-Scl-70	Anti-DNA topoisomerase I antibodies
anti-Sm	Anti-Smith antigen antibodies
anti-SSA	Anti-Sjögren’s-syndrome-related antigen A antibodies
anti-SSB	Anti-Sjögren’s-syndrome-related antigen B antibodies
anti-TPO	Anti-thyroid peroxidase antibody
BAFF	B cell-activating factor
BMI	Body mass index
CENP-B	Centromere protein B
DNA	Deoxyribonucleic acid
ELISA	Enzyme-linked immunosorbent assay
FSH	Follicle-stimulating hormone
HEp-2	Human epithelial type 2 cells
HLA	Human Leukocyte Antigen
IF	Immunofluorescence
IIF	Indirect immunofluorescence
IL-6	Interleukin 6
IL-17	Interleukin 17
IL-23	Interleukin 23
IQR	Interquartile range
IU/mL	International units per milliliter
Jo-1	Histidyl-tRNA synthetase
LH	Luteinizing hormone
NETs	Neutrophil extracellular traps
NIH	National Institutes of Health
NK	Natural killer
NOS	Newcastle–Ottawa Scale
OD	Optical density
OGTT	Oral glucose tolerance test
PCOS	Polycystic ovary syndrome
PRISMA	Preferred Reporting Items for Systematic reviews and Meta-Analyses
PROSPERO	International Prospective Register of Systematic Reviews
RNA	Ribonucleic acid
RNP/Sm	Ribonucleoprotein/Smith complex antigen
RNP70	Ribonucleoprotein 70
SARDs	Systemic autoimmune rheumatic diseases
Scl-70	DNA topoisomerase I
Sm	Smith antigen
SSA	Sjögren’s-syndrome-related antigen A
SSB	Sjögren’s-syndrome-related antigen B
Th1	Type 1 T helper
Th17	Type 17 T helper
TNF-α	Tumor necrosis factor α
Treg	Regulatory T cells
TSH	Thyroid-stimulating hormone
