# Supplementary material for: Antinuclear Antibodies in Polycystic Ovary Syndrome: A Systematic Review of Observational Studies
Source: Int J Mol Sci. 2025 Sep 28;26(19):9493. doi: 10.3390/ijms26199493 (PMC12529972; doi:10.3390/ijms26199493)
Supplement: Supplementary file 1 [file ijms-26-09493-s001.zip › Supplementary material S1 – search strategy.pdf]

SUPPLEMENTARY MATERIAL S1 – SEARCH STRATEGY

A comprehensive literature search was performed on August 9, 2025, using three major databases: PubMed, Embase, and Scopus. The aim was to identify observational studies evaluating the presence or levels of autoantibodies in women with polycystic ovary syndrome (PCOS). The search strategy included terms related to autoimmunity and PCOS, applied to the title, abstract, keywords, or MeSH terms (where applicable). No date or language restrictions were applied.

The full electronic search strategies for each database were as follows:

PubMed (n = 390)

(("anti nuclear"[Title/Abstract]) OR ("anti-nuclear"[Title/Abstract]) OR (antinuclear[Title/Abstract]) OR (ANA[Title/Abstract]) OR (antibodies, antinuclear[MeSH Terms]) OR (antibody, antinuclear[MeSH Terms]) OR (antinuclear antibodies[MeSH Terms]) OR (antinuclear antibody[MeSH Terms]) OR ("anti-dsDNA"[Title/Abstract]) OR ("anti-histone"[Title/Abstract]) OR ("anti-Ro"[Title/Abstract]) OR ("anti-La"[Title/Abstract]) OR ("anti-Sm"[Title/Abstract]) OR ("anti-RNP"[Title/Abstract]) OR (antibodies[Title/Abstract]) OR (autoantibodies[Title/Abstract]) OR (autoimmunity[Title/Abstract]) OR (autoimmune[Title/Abstract]) OR ("autoimmune antibodies"[Title/Abstract]) OR ("self-antibodies"[Title/Abstract]) OR ("autoreactive antibodies"[Title/Abstract]) OR ("autoantigen antibodies"[Title/Abstract]) OR (autoimmunization[Title/Abstract]) OR ("immune dysregulation"[Title/Abstract]) OR ("autoimmune disease"[Title/Abstract]) OR (autoantibodies[MeSH Terms]) OR (autoimmunity[MeSH Terms])) AND ((polycystic ovary syndrome[Title/Abstract]) OR ("polycystic ovarian syndrome"[Title/Abstract]) OR (PCOS[Title/Abstract]) OR ("Stein-Leventhal syndrome"[Title/Abstract]) OR ("polycystic ovary disease"[Title/Abstract]) OR ("polycystic ovarian disease"[Title/Abstract]) OR ("sclerocystic ovary syndrome"[Title/Abstract]) OR (PCOD[Title/Abstract]) OR (polycystic ovarian syndrome[MeSH Terms]) OR (polycystic ovary syndrome[MeSH Terms]) OR (syndrome, polycystic ovary[MeSH Terms]))

Embase (via Elsevier) (n = 707)

('anti nuclear':ti,ab,kw OR 'anti-nuclear':ti,ab,kw OR antinuclear:ti,ab,kw OR ANA:ti,ab,kw OR 'anti-dsDNA':ti,ab,kw OR 'anti-histone':ti,ab,kw OR 'anti-Ro':ti,ab,kw OR 'anti-La':ti,ab,kw OR 'anti-Sm':ti,ab,kw OR 'anti-RNP':ti,ab,kw OR antibodies:ti,ab,kw OR autoantibodies:ti,ab,kw OR autoimmunity:ti,ab,kw OR autoimmune:ti,ab,kw OR 'autoimmune antibodies':ti,ab,kw OR 'self-antibodies':ti,ab,kw OR 'autoreactive antibodies':ti,ab,kw OR 'autoantigen antibodies':ti,ab,kw OR autoimmunization:ti,ab,kw OR 'immune dysregulation':ti,ab,kw OR 'autoimmune disease':ti,ab,kw) AND ('polycystic ovary syndrome':ti,ab,kw OR 'polycystic ovarian syndrome':ti,ab,kw OR pcos:ti,ab,kw OR 'stein-

leventhal syndrome':ti,ab,kw OR 'polycystic ovary disease':ti,ab,kw OR 'polycystic ovarian disease':ti,ab,kw OR 'sclerocystic ovary syndrome':ti,ab,kw OR pcod:ti,ab,kw)

Scopus (n = 763)

TITLE-ABS-KEY ("anti nuclear" OR "anti-nuclear" OR antinuclear OR ANA OR "anti-dsDNA" OR "anti-histone" OR "anti-Ro" OR "anti-La" OR "anti-Sm" OR "anti-RNP" OR antibodies OR autoantibodies OR autoimmunity OR autoimmune OR "autoimmune antibodies" OR "self-antibodies" OR "autoreactive antibodies" OR "autoantigen antibodies" OR autoimmunization OR "immune dysregulation" OR "autoimmune disease") AND ("polycystic ovary syndrome" OR "polycystic ovarian syndrome" OR pcos OR "Stein-Leventhal syndrome" OR "polycystic ovary disease" OR "polycystic ovarian disease" OR "sclerocystic ovary syndrome" OR pcod)
