# Supplementary material for: Antinuclear Antibodies in Polycystic Ovary Syndrome: A Systematic Review of Observational Studies
Source: Int J Mol Sci. 2025 Sep 28;26(19):9493. doi: 10.3390/ijms26199493 (PMC12529972; doi:10.3390/ijms26199493)
Supplement: Supplementary file 1 [file ijms-26-09493-s001.zip › Supplementary material S2 – modified Newcastle-Ottawa quality assessment scale.pdf]

SUPPLEMENTARY MATERIAL S2: MODIFIED NEWCASTLE-OTTAWA QUALITY ASSESSMENT SCALE

(Adapted to assess observational studies evaluating antinuclear autoantibodies in women with PCOS)

Note: A study can be awarded a maximum of one star (★) for each criterion fulfilled within the Selection, Comparability, and Outcome domains, unless otherwise specified.

I. SELECTION DOMAIN ***(Maximum score: 4 stars)***

1. **Use of Internationally Recognized PCOS Definition**
   Yes: Internationally recognized diagnostic criteria for PCOS (e.g., Rotterdam, NIH) were clearly stated and applied. ★
   No: No PCOS definition was provided, or a non-standard definition was used.
2. **Clearly Defined and Appropriate Inclusion/Exclusion Criteria**
   Yes: Inclusion and exclusion criteria for the PCOS group were clearly stated and aligned with the diagnostic criteria used (e.g., Rotterdam, NIH), ensuring appropriate selection of participants. ★
   No: Criteria were not stated, incomplete, or not aligned with established diagnostic standards.
3. **Proper Selection and Description of a Control Group**
   Yes: The control group consisted of women without PCOS and was clearly defined with appropriate criteria (e.g., disease-free, reproductive age, regular menstrual cycles, no signs of hyperandrogenism, normal ovarian morphology, and normal hormone levels), and adequately described. ★
   No: Control group selection or health status description was unclear or insufficient.
4. **Use and Reporting of a Validated Method for Autoantibody Measurement**
   Yes: A validated method for autoantibody measurement was clearly reported (e.g., enzyme-linked immunosorbent assay [ELISA], indirect immunofluorescence [IIF]), including reference ranges or assay details. ★
   No: The method for autoantibody measurement was not reported, insufficiently described, or not validated.

II. COMPARABILITY DOMAIN ***(Maximum score: 2 stars)***

1. **Age Matching or Statistical Control**
   Yes: Age was matched or statistically controlled for between PCOS and control groups. ★
   No: Age was not matched or controlled for.
2. **Matching or Control for BMI**
   Yes: BMI was matched or statistically controlled for between groups. ★
   No: BMI was not matched or controlled for.

III. OUTCOME DOMAIN ***(Maximum score: 3 stars)***

1. **Clarity of Outcome Presentation**
   Yes: Outcomes (e.g., prevalence or levels of autoantibodies) were clearly reported, with consistent and unambiguous presentation (e.g., clear distinction between positive and negative cases, no discrepancies between text and tables). ★
   No: Outcome data were presented unclearly, inconsistently, or lacked key information needed for interpretation.

2. **Consideration of Potential Confounders**
   Yes: The study clearly addressed key confounders influencing autoantibody levels, particularly by including excluding individuals with a history of systemic/autoimmune/chronic disorders or ANA-inducing drugs or aromatase inhibitors. ★
   No: Potential confounders were not adequately considered or controlled for.

3. **Appropriate Statistical Methods**
   Yes: Statistical methods used to analyze the outcomes (e.g., prevalence or levels of autoantibodies) were appropriate and clearly reported. ★
   No: Statistical methods were inappropriate, unclear, or not adequately reported.

Risk of Bias Interpretation:

Studies were categorized as low risk of bias (7–9 stars), moderate risk (4–6 stars), and high risk (0–3 stars).

Adapted from the Newcastle-Ottawa Scale for assessing the quality of nonrandomized studies in meta-analyses. Available at: https://www.ohri.ca/programs/clinical_epidemiology/oxford.asp
